# Femoral artery pseudoaneurysm secondary to percutaneous femoral artery intervention: a case report

**DOI:** 10.1093/jscr/rjag128

**Published:** 2026-03-21

**Authors:** Ying Chen

**Affiliations:** Department of Vascular Surgery, Union Hospital, Tongji Medical College, Huazhong University of Science and Technology, No. 1277 Jiefang Avenue, Wuhan 430022, China

**Keywords:** pseudoaneurysm, intervention complication, artery injury, open surgery, case report

## Abstract

The clinical presentation, interventional strategy, and outcomes of a single case of femoral artery pseudoaneurysm (FAP) were analysed. There was clear evidence of ischemia and infection in the right groin. The patient was successfully treated with open surgery combined with arterial bypass. The FAP was successfully removed, and no postoperative extremity ischemia was observed. Open surgery could be an effective method for managing FAP, with or without infection.

## Introduction

Percutaneous femoral artery puncture is the most commonly used method of access for endovascular interventions. Haematoma, pseudoaneurysm, and arterial occlusion are the most frequent local complications related to the femoral arterial puncture site [1]. Early reports showed that femoral artery pseudoaneurysms (FAPs) occur in 0.8%-2.2% of the population following interventional treatments [2]. Endovascular therapy is becoming a first-line treatment for various vascular diseases, with increasing attention given to FAP as a potential complication. Recent reports demonstrate that, even with routine ultrasound (US)-guided arterial puncture, the incidence of FAP is ~2.9% [3]. A pseudoaneurysm, also known as a false aneurysm, is typically characterized by pulsatile hematoma contained within a layer of fibrin and surrounding tissues [4]. Unlike real aneurysms, pseudoaneurysms are contained either only by the media and adventitia or by the surrounding tissue. Most uncomplicated FAPs can be cured without open surgery, either through observational follow-up or using US-guided or endovascular techniques to close the lesion. For complicated FAPs and those failing nonsurgical treatment, surgical intervention is needed to close the pseudoaneurysm [5]. This case report demonstrates an example of a severe FAP complication following neurointervention through the right femoral artery access.

## Case presentation

**Figure 1:**
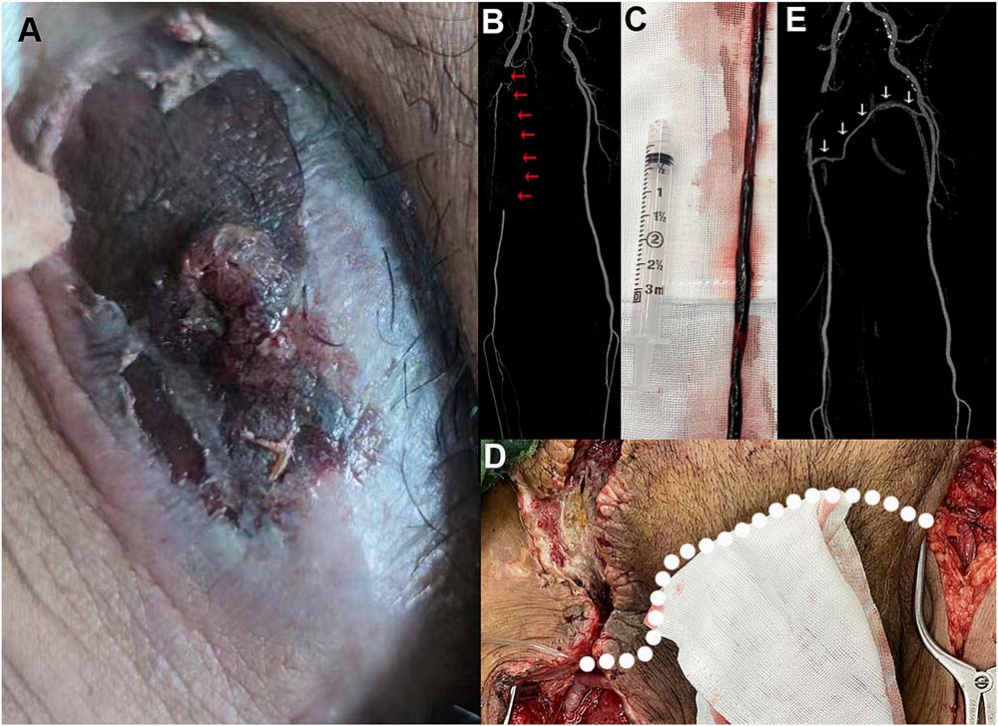
Representative images of femoral artery pseudoaneurysm. (A) A preoperative pulsatile hematoma in the right groin. (B) CT image with the red arrows pointing to the obstructive superficial femoral artery. (C) The removed arterial thrombosis. (D) Intraoperative bypass of left-to-right femoral artery; white dashed line showing the autologous vein trajectory. (E) Postoperative computed tomography angiography image showing the autologous vessel (white arrows) and the right extremity demonstrating patent arterial flow.

A 68-year-old male patient (body mass index = 26) with painful pulsatile hematoma in the right groin presented to the vascular surgery department (Fig. 1A). He had undergone endovascular embolization of a cerebral aneurysm 2 weeks prior at an external facility. The patient complained of numbness in the right foot. There was no pulsation in the foot artery and posterior tibial artery of the right lower limb. On presentation, his temperature was 98.2°F (36.8°C) blood pressure 135/70 mm Hg, heart rate 88/min, and oxygen saturation 99% on room air. His white blood cell count was 15.1 × 10^9^/L (reference range, 3.8–9.8 × 10^9^/L), and manual differential revealed 81.2% eosinophils (absolute eosinophil count, 12.3 × 10^9^/L) and no blasts. Additionally, his haemoglobin level was 84 g/L (reference range, 130-175 g/L), with a normal platelet count, and his D-dimer level was 1.5 μg/ml (8.21 nmol/L) (reference range, 0.0–0.99 μg/ml [0.0-5.42 nmol/L]). Subsequently, computed tomography angiography (CTA) showed an embolism in the right femoral artery (Fig. 1B, red arrows). As there was obvious ischemia in the right extremity, emergency surgery was performed under general anesthesia. After dissecting the right groin area, the hematoma was removed. However, in addition to the FAP, there was also clear evidence of infection and femoral artery thrombosis. Thrombectomy of the superficial femoral artery was then performed (Fig. 1C). Due to severe infection and intraoperative bleeding, the right common femoral artery was ligated. To address the right-leg ischemia, a left-to-right femoral–femoral bypass was performed using an autogenous great saphenous vein vessel (Fig. 1D, white dashed line). The postoperative CTA showed the patency of the artery bypass tunnel (Fig. 1E, white arrows). Before his discharge, the pulsatile hematoma disappeared in the right groin. At the 3-month follow-up, the patient was able to walk freely without intermittent claudication.

## Discussion

As a common disease in vascular surgery, FAP is mainly caused by trauma and iatrogenic factors. At present, the treatment methods for iatrogenic FAP mainly include US-guided compression, US-guided local thrombin injection therapy, and surgical intervention, among others [1]. If the disease is not treated properly at an early stage, local aneurysms and surrounding haematomas are prone to infection, forming infectious aneurysms. Infectious aneurysms not only cause poor local wound healing and skin necrosis but also increase the risk of aneurysm rupture, bleeding, and haemorrhagic shock. The formation and detachment of the thrombus in the pseudoaneurysm cavity can lead to distal arterial embolism, as shown in the case presented here, and infection circulating with the blood may further cause bacteremia, affecting the whole body. The patient's condition lasted for ~2 weeks before presenting to our hospital, and he had not received proper targeted treatment. Through blood tests and an intraoperative exploration of the wound after admission, it was confirmed that the patient had an infectious FAP. The basic surgical methods for managing infectious aneurysms include thorough debridement and vascular repair. Because infected wounds and local skin defects often cannot be sutured in one stage, frequent changes in wound dressings after surgery are required, which is not conducive to controlling wound infections and can easily lead to secondary infections. The closed negative pressure-assisted closure device can cover the wound for about a week, and continuous negative pressure drainage helps to control infection, providing an effective treatment method for postoperative infectious wound treatment [6]. To avoid the formation of iatrogenic FAP, emphasis should be placed on improving the operator's operational skills, including US-guided puncture, puncture techniques, selection of puncture site, and whether local compression is thorough after puncture [7]. Based on the patient's own condition, including obesity, severe atherosclerotic plaques at the local puncture site, poor control of hypertension and the use of postoperative anticoagulants and antiplatelet drugs, targeted preoperative prevention are necessary.

## Limitations

There are several limitations to this study. First, we reported a single case, which inherently limits generalizability and may introduce bias in the discussion. Another weakness is that the femoral artery procedure for the case presented was conducted without US assistance, which is the technique commonly used in endovascular therapy.

## Conclusions

For pseudoaneurysms that have already formed, timely diagnosis is essential, and treatment should be implemented based on the progression of the disease. Once a pseudoaneurysm becomes infected, open surgery should be performed promptly to control wound inflammation and to select the appropriate surgical methods based on the extent of arterial injury.

## Data Availability

The datasets used and analysed during the current study are available from the corresponding author on reasonable request.
